# Stress tolerant virulent strains of *Cronobacter sakazakii* from food

**DOI:** 10.1186/0717-6287-47-63

**Published:** 2014-11-25

**Authors:** Md Fakruddin, Mizanur Rahaman, Monzur Morshed Ahmed, Md Mahfuzul Hoque

**Affiliations:** Industrial Microbiology Laboratory, Institute of Food Science and Technology (IFST), Bangladesh Council of Scientific and Industrial Research (BCSIR), Dhaka, Bangladesh; Department of Microbiology, University of Dhaka, Dhaka, Bangladesh

**Keywords:** *Cronobacter*, Food, Virulent, Bangladesh

## Abstract

**Background:**

*Cronobacter sakazakii* is considered as an emerging foodborne pathogen. The aim of this study was to isolate and characterize virulent strains of *Cronobacter sakazakii* from food samples of Bangladesh.

**Result:**

Six (6) *Cronobacter sakazakii* was isolated and identified from 54 food samples on the basis of biochemical characteristics, sugar fermentation, SDS-PAGE of whole cell protein, plasmid profile and PCR of *Cronobacter* spp*.* specific genes (*esak*, *gluA*, *zpx*, *ompA*, ERIC, BOX-AIR) and sequencing. These strains were found to have moderately high antibiotic resistance against common antibiotics and some are ESBL producer. Most of the *C. sakazakii* isolates were capable of producing biofilm (strong biofilm producer), extracellular protease and siderophores, *curli* expression, haemolysin, haemagglutinin, mannose resistant haemagglutinin, had high cell surface hydrophobicity, significant resistance to human serum, can tolerate high concentration of salt, bile and DNase production. Most of them produced enterotoxins of different molecular weight. The isolates pose significant serological cross-reactivity with other gram negative pathogens such as serotypes of *Salmonella* spp., *Shigella boydii*, *Shigella sonnei*, *Shigella flexneri* and *Vibrio cholerae*. They had significant tolerance to high temperature, low pH, dryness and osmotic stress.

**Conclusion:**

Special attention should be given in ensuring hygiene in production and post-processing to prevent contamination of food with such stress-tolerant virulent *Cronobacter sakazakii*.

**Electronic supplementary material:**

The online version of this article (doi:10.1186/0717-6287-47-63) contains supplementary material, which is available to authorized users.

## Background

*Cronobacter sakazakii* is an opportunistic foodborne pathogen associated with infections in neonates and infants; particularly those that are premature or immune compromised [1]. Symptoms of *Cronobacter sakazakii* infection are severe, including meningitis, septicemia and necrotizing enterocolitis [2]. The original reservoir of *C. sakazakii* is still unknown [3] but The organism is ubiquitous in nature and *C. sakazakii* has been recovered from powdered infant milk formula (PIF) in a number of countries throughout the world [4] and contaminated PIF has been epidemiologically linked with several cases of *C. sakazakii* infections in infants [5]. *Cronobacter sakazakii* has been isolated from various food products such as mixed salad vegetables, meat, milk and cheese [6].

Low birth-weight neonates (i.e. <2.5 kg) and infants of <28 days age are at heightened risk compared to more mature infants [2]. Symptoms include meningitis leading to ventriculitis, brain abscess, hydrocephalus and cyst formation as well as necrotizing enterocolitis characterized by intestinal necrosis and pneumatosis intestinalis; pulmonary, urinary and blood stream infections [7]. The mortality rate for neonatal infections has been reported to be as high as 80% [8] and survivors often suffer from severe irreversible neurological disorders. Food other than infant formula has been rarely investigated for the presence of *C. sakazakii.* Nevertheless, this microorganism could be isolated from a wide spectrum of food and food ingredients.

Identification of virulence factors is important in understanding bacterial pathogenesis and their interactions with the host, which may also serve as novel targets in drug and vaccine development [9].Virulence factor of *Cronobacter sakazakii* is the O antigen, production of proteolytic enzymes etc. Virulence factors and mechanisms of *Cronobacter sakazakii* still not elucidated fully and *C. sakazakii* isolated from different regions may differ in their virulence properties.

Data on the presence and virulence properties of *Cronobacter sakazakii* in food consumed among children of Bangladesh are still not reported. Thus the present study aimed to detect the presence of virulent strains of *Cronobacter sakazakii* from food samples of Bangladesh.

## Results

### Isolation and identification of *Cronobacter sakazakii*

A total of 54 isolates have been screened primarily and six isolates were identified as *Cronobacter sakazakii*. All the six isolates (MP04.1, MP08.5, MP10.2, HR11.3, BC 52.2 & SP62.1) produced characteristic red/pink colonies on VRVG agar (Oxoid, UK) and yellow pigmentation and water like yellow pigmentation on TSA respectively (Additional file 1). All the isolates pose similar biochemical characteristics as *Cronobacter sakazakii* such as oxidase negative, catalase positive, citrate positive, MR-VP and nitrate reduction negative. All the six isolates capable to ferment glucose and lactose on KIA, motile, indole positive, can decarboxylate arginine and hydrolyse esculin and liquefy gelatin. The isolates vary in their sugar fermentation pattern. All of them were unable to ferment dulcitol and malonate and capable to ferment rhamnose, xylose, trehalose, arabinose, cellubiose, melibiose. Salicin, maltose and sorbitol fermented by 3 isolate each and mannitol, glucose and sucrose femrneted by 4 isolates each whereas lacotose fermented by 2 isolates. All of them showed fluorescence under UV light (250 nm) on MUG-MacConkey agar and produced “Blue- Green” colonies on HicromeEnterobacter sakazakii agar (HiMedia, India) because of the production of α-glucosidase enzyme.

### SDS-PAGE analysis of whole cell proteins

*Cronobacter muytjensii* ATCC 51329 and *Cronobacter sakazakii* ATCC 29544 shared similar molecular weight protein bands (10KDa & 25KDa) with the isolates (Table 1). Similarities of whole cell proteins among isolates, *C. sakazakii* ATCC 29544 and *C. muytjensii* ATCC 51329 justify their identity as *Cronobacter sakazakii*.

**Table 1:** **Approximate molecular weights (MW) of whole cell proteins extracted from presumptive isolates of** ***Cronobacter*** **spp. naked eye visualization comparison with marker**

Sample ID	MW of standard protein bands (KDa)
**C. muytjensii ATCC 51329**	10,25, 28, 35, 40,80,140,150,160 & 220
**C. sakazakii ATCC 29544**	10,25, 28, 35, 80, 150,160 & 220
**MP04.1**	10,25, 27 & 35
**MP10.2**	10,25, 26, 35,40,150,200 & 220
**CL41.1**	10,25, 100 & 225
**CL41.2**	10,26,28 & 42
**BC52.2**	10,15,20 25, 35,42, 50,70,110,150 & 200
**BC59.2**	10,25 27, 32,40,70,100,150 & 200
**SP62.1**	10,25, 70 &140

### Plasmid profiling of the isolates

All the isolates pose a common plasmid (molecular weight ≥2 kb) similar to *Cronobacter muytjensii* ATCC 51329. Two of the isolates also pose additional plasmid (molecular weight ≤2 kb).

### Molecular detection of the isolates through PCR amplification

Results of the PCR detection methods, using primers reported as specific for *C. sakazakii* are summarized in Table 2. Desirable PCR product (929 bp) of Esakf/Esakr primer pair was obtained in all the isolates and the type strain *Cronobacter muytjensii* ATCC 51329. Desirable PCR product (1680 bp) of EsgluAf/EsgluAr primer pair was obtained in isolate MP 08.5 and the type strain Cronobacter sakazakii ATCC 29554. Desirable PCR product (952 bp) for *saka* gene was obtained in three isolates (MP04.1, HR11.3 & BC52.2) and the type strain *C. muytjensii* ATCC 51329

**Table 2:** **Results of** ***Cronobacter sakazakii*** **specific PCR using different primers**

Isolates ID	Source	Primer pairs used				
		Esakf/Esakr	EsgluAf/EsgluAr	Saka1a/Saka2b	ESSF/ESSR	ZpxF/ZpxR
*C. muytjensii*	ATCC 51329	+	-	+	+	-
*C. sakazakii*	ATCC 29544	+	+	+	+	+
MP 04.1	Milk powder	+	-	+	-	-
MP 08.5	Milk powder	+	+	-	-	-
MP 10.2	Milk powder	+	-	-	-	-
HR 11.3	Horlicks	+	-	+	-	-
BC 52.2	Biscuit	+	-	+	-	+
SP 62.1	Spice	+	-	-	+	+

### 16 s rDNA sequencing

DNA sequencing of 16 s rDNA of the isolates showed more than 90% resemblance with sequences of *Cronobacter sakazakii* deposited in database (NCBI) and is thus confirmed as *Cronobacter sakazakii*. Identification by 16 s rDNA sequencing and BLAST with accession numbers are presented in Table 3.

**Table 3:** **16s rDNA sequencing result of the isolates**

Sl no	Isolate no	Identification	Accssion number
1	MP04.1	*Cronobacter sakazakii*	KC818225.1
2	MP08.5	*Cronobacter sakazakii*	FJ906924.1
3	MP10.2	*Cronobacter sakazakii*	KC990826.1
4	HR11.3	*Cronobacter sakazakii*	JQ963912.1
5	BC52.2	*Cronobacter sakazakii*	KC818229.1
6	SP62.1	*Cronobacter sakazakii*	FN401361.1

### Phylogeny

**Figure 1:**
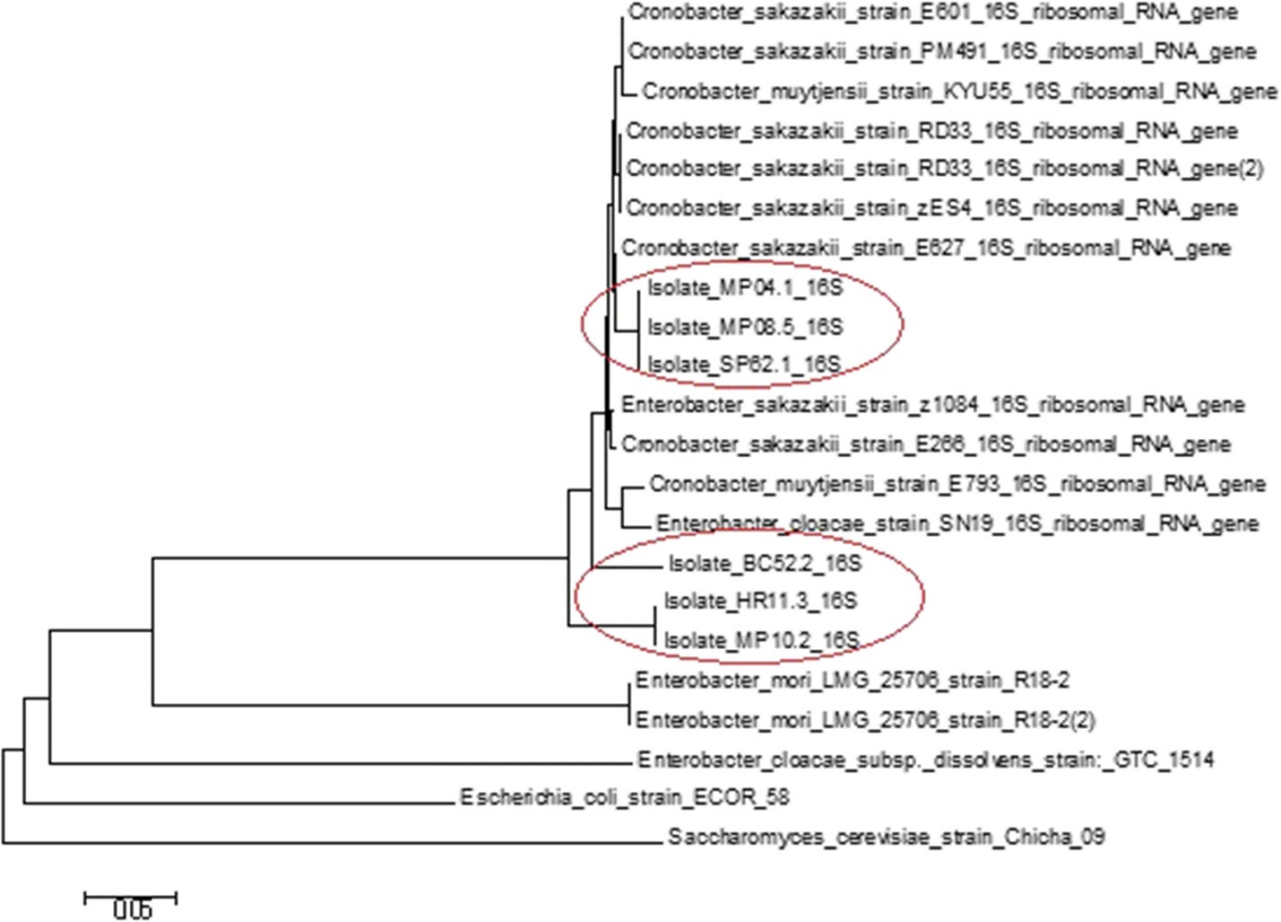
**Evolutionary relationships of the isolated** ***Cronobacter sakazakii*** **with related species.**

The 6 isolates were found in two different clusters in the phylogenetic tree. Isolates MP04.1, MP08.5 & SP62.1 in a cluster and isolates BC52.2, HR11.3 & MP10.2 in another cluster (Figure 1). All the isolates are phylogeneticcally closely linked with *Cronobacter sakazakii* stains reported earlier present in databases. Phylogenetic analysis by MEGA 5 reveals that the 6 isolates were phylogenetically different position in different place in the tree.

### BOX-PCR

In BOX PCR all the isolates have some common yet some different fingerprint than *C. muytjensii* ATCC 51329 and C. sakazakii ATCC 29544 which may be due to differ in species level. All the isolates have 5 common bands in BOX-PCR fingerprint which supports this identity as of same species (*C. sakazakii*). These isolates also have significant differences in this BOX fingerprint which indicates that these isolates belong to different genotypes of *C. sakazakii*. These isolates also differ in this isolation habitat which may also contribution to this differ in BOX fingerprints [10].

### ERIC-PCR for isolated strains

In ERIC-PCR *C. muytjensii* ATCC 51329 and *C. sakazakii* ATCC 29544 produced two (2) major bands (200 bp & 400 bp). No prominent band was observed for isolate MP04.1 Two (2) prominent bands (1000 bp & 800 bp) were observed for isolate MP08.5. Four (4) prominent bands (380 bp, 500 bp, 700 bp & 850 bp) were observed in isolate MP10.2. Two (2) prominent bands (650 bp& 820 bp) were observed in isolate HR11.3. Two (2) major bands (750 bp & 820 bp) were observed in isolate BC52.2. Six (6) major bands were observed in isolate SP62.1 ranging from 400 bp to larger than 2 kb. 750 bp was found in 3 isolated *Cronobacter sakazakii* (HR11.3, BC52.2 & SP62.1). 820 bp was found in 2 *Cronobacter sakazakii* (HR11.3 & BC52.2). Variations in ERIC-PCR product also demonstrate that all the 6 isolated *Cronobater sakazakii* strains belong to different genotypes.

### Source analysis of isolated *Cronobacter sakazakii*

Source wise analysis reveals that milk powder (27.78%), horlicks (11.11%), biscuits (18.52%), and spices (9.26%) samples were contaminated with *C. sakazakii*. 3 (50%) strains of *C. sakazakii* isolated from milk powder, 1(16.67%) from horlicks, 1(16.67%) from biscuits and 1(16.67%) from spices samples. None of the honey, Chutney & chocolate samples contaminated with *C. sakazakii* through a number of phenotypically (not genotypically) similar organisms were found in these samples.

### Antibiotic susceptibility patterns of the isolates

**Figure 2:**
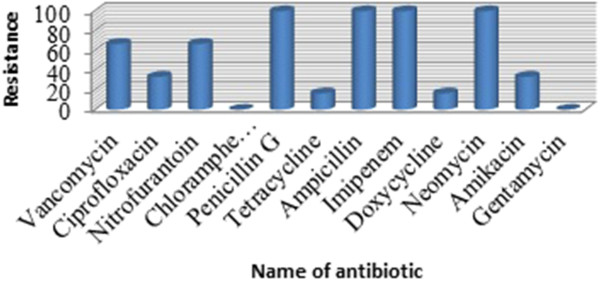
**Antibiotic resistance pattern of isolated** ***Cronobacter sakazakii.***

All of the 6 isolated *cronobacter sakazakii* were sensitive to chloramphenicol, gentamycin and most of them were resistant to vancomycin, ampicillin, nitrofurantoin, penicillin G, imipenem, In this study only one strain (SP62.1) was resistant to tetracycline and doxycycline (Figure 2). 2 of the 6 isolates showed ESBL activity.

### Virulence properties of isolated *Cronobacter sakazakii*

50% (3/6) isolated *Cronobacter sakazakii* was found to be able to produce protease on skim milk agar. 50% (3/6) isolates were able to bind congo red indicating curli expression. 40% (2/6) isolates showed hemolysis activity on human blood agar. All isolates were haemagglutination positive and mannose-resistant haemagglutination (MRHA) positive on slide-agglutination test. Four (4) *Cronobacter sakazakii* strains (66.67%) aggregated with 3% (NH_4_)_2_SO_4_ solution, BC52.2 and SP62.1 both had no aggregation with 3% (NH_4_)_2_SO_4_ solution. *Cronobacter muytjensii* ATCC 51329 also aggregated with the same concentration. All the six isolated *C. sakazakii* were able to produce siderophore, an important virulence factor of bacterial pathogens. Virulence properties of the *C. sakazakii* isolates are shown in Table 4.

**Table 4:** **Some virulence properties of isolated of** ***Cronobacter sakazakii***

Sample ID	PPA	CRB	Hem	HG	MS	BCSH	SD
**C. muytjensii ATCC 51329**	+	+	-	+	+	+	+
**C. sakazakii ATCC 29544**							
**MP04.1**	-	+	-	+	+	+	+
**MP08.5**	+	+	+	+	+	+	+
**MP10.2**	-	-	+	+	+	+	+
**HR11.3**	-	-	-	+	+	+	+
**BC52.2**	+	-	-	+	+	-	+
**SP62.1**	+	+	-	+	+	-	+

(PPA = Protease Production Activity, CRB = Congo Red Binding Capability, Hem = Blood hemolysis, HG = Haemagglutination, MS = Mannose-sensitivity, BCSH = Bacterial cell surface hydrophobicity, SD = Siderophore production).

**Figure 3:**
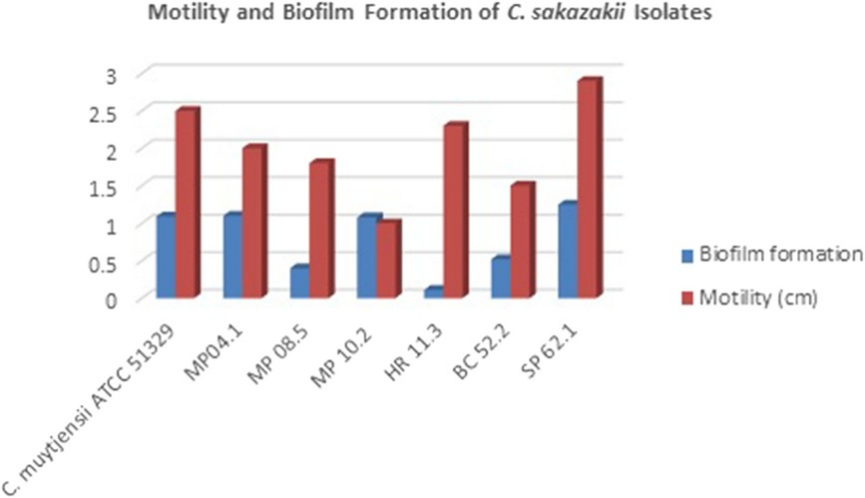
**Comparative results between biofilm formation and motility among isolated** ***Cronobacter sakazakii.***

All isolated strains along with *C. muytjensii* ATCC 51329 were tested for their serum tolerance. About 50% colonies were reduced after serum treatment. A total of 6 isolates were taken for the biofilm assay. All the isolates gave significant results. Three (MP04.1, MP10.2 & SP62.1) of them were found to capable of biofilm production on polystyrene microtiter plate (Figure 3).

### SDS-PAGE analysis of enterotoxin of the isolates

All of the six *Cronobacter sakazakii* were produced enterotoxin along with protein of different molecular weight. Type strains *C. muytjensii* ATCC 51329 and *C. sakazakii* ATCC 29544 was used as a positive control (Table 5). Ammonium sulfate precipitation was applied to purify the enterotoxin. 50% of salts were applied to the cell free supernatant, which significantly affected the precipitation of enterotoxin production.

**Table 5:** **Molecular weights (MW) of enterotoxin extracted from** ***C. sakazakii*** **isolates**

Sample ID	MW of standard protein bands (KDa)
***C. muytjensii*** **ATCC 51329**	66, 80 & 150
***C. sakazakii*** **ATCC 29544**	66, 125
**MP04.1**	66 & 80
**MP08.5**	66, 80,125 & 150
**MP10.2**	150
**HR11.3**	66, 80,125 & 150
**BC52.2**	66, 80,125 & 150
**SP62.1**	66, 80,125 and 150

### Detection of virulence genes (ompA & zpx)

Two isolate (SP62.1 & MP 10.2) and the type strain *C. muytjensii* ATCC 51329 and *C. sakazakii* ATCC 29544 produced desirable PCR product (469 bp) for *ompA* gene. The outer membrane protein A (OmpA) of *Cronobacter sakazakii* is involved in the colonization of the gastrointestinal tract and invasion of human intestinal epithelial and brain endothelial cells, as well as subsequent survival in blood to cause meningitis [11, 12]. Desirable PCR product (94 bp) for zpx gene was obtained in BC52.2 & P62.1. Zinc-containing metalloprotease encoded by *zpx* gene are produced by a number of pathogenic bacteria. Specific degradation of extracellular matrix protein components, such as type intravenous (IV) collagen, may cause destruction of endothelial cell membranes of capillary vessels, leading to the leakage of blood components into surrounding tissues, thus enabling pathogens to cross the blood–brain barrier Mohan *et al*. [11].

### Serological cross reactivity of the *Cronobacter sakazakii*isolates

Antigenic determinants expressed on the bacterial cell surface are of importance in the serological characterization and microbiological diagnosis. The bacterial strains carrying these identical or similar antigenic epitopes might react with antibodies produced against other strains. Serological cross reactivity between different groups of pathogens has been reported earlier. To determine whether the isolated *Cronobacter sakazakii* strains have any serological cross reactivity, slide agglutination was performed against commercial antisera of different gram negative bacteria. The isolates showed significant serological cross reactivity with different serotypes of *Salmonella*, *Shigella boydii* and *Vibrio cholerae*. Results of the serological cross reactivity have been showed in Table 6.

**Table 6:** **Different antisera agglutination test for isolated** ***Cronobacter sakazakii*** **strains**

Sample ID	1	2	3	4	5	6	7	8	9	10	11	12	13	14
**MP04.1**	+	+	-	-	-	+	-	+	-	-	-	+	-	-
**MP08.5**	-	+	-	+	-	+	-	-	-	-	+	-	-	-
**MP10.2**	+	+	+	+	+	+	+	+	+	+	-	+	+	+
**HR11.3**	-	-	+	-	+	-	-	-	-	-	+	-	-	-
**BC52.2**	-	+	-	+	-	-	+	-	-	-	-	+	-	-
**SP62.1**	+	-	+	+	-	-	+	-	-	-	-	-	-	-

1. *Salmonella* 2*–*0, 2. *Salmonella* polyvalent O group A-S, 3. *Salmonella typhi* O-Group D somatic antigen, 4. *Salmonella paratyphi* A O Group A somatic antigen, 5. *Salmonella* 9*–*0, 6. *Shigella boydii* polyvalent 3(12–13) 7. *Shigella boydii* polyvalent 1(1–6), 8. *Shigella sonnei* phase 1&2, 9. *Shigella flexneri* polyvalent (1–6, x & y), 10. *Shigella boydii* polyvalent 2(7–11), 11. *Shigella boydii* polyvalent 3(2–15), 12. *Vibrio cholerae* O1 polyvalent, 13. *Vibrio cholerae* inaba, 14. *Vibrio cholerae* ogawa.

### Stress tolerance

#### Salt tolerance test

Four (66.67%) isolated *Cronobacter sakazakii* were able to grow at 10% NaCl concentration. MP04.1 and HR11.3 both were able to grow at 7%.

#### Bile salt tolerance

All of isolated (6/6) *Cronobacter sakazakii* were able to grow 5% bile salt concentration.

#### Thermotolerance

D-values for the isolated *C. sakazakii* strains, suspended in TSB and IFM, were determined from 54 to 62°C (Table 7). At 54°C the D-values ranged from 15.75 (±0.18) to 18.24 (±0.21) min. The D-values were between 0.57 (±0.21) and 1.12 (±0.19) min when the treatment temperature was raised to 62°C. Z-value for the isolates ranged from 6.4 (±0.14) and 6.7 (±0.18).

**Table 7:** **Thermotolerance (D-values and z-values) of isolated** ***C. sakazakii*** **(SP 62.1)**

Medium	D-value (min)					z-value (°C)
	54°C	56°C	58°C	60°C	62°C	
TSB	15.75 ± 0.18	7.54 ± 0.11	3.84 ± 0.32	1.64 ± 0.17	0.57 ± 0.21	6.4 ± 0.14
IFM	18.24 ± 0.21	9.32 ± 0.08	5.11 ± 0.24	2.35 ± 0.25	1.12 ± 0.19	6.7 ± 0.18

TSB = Trypticase soy broth; IFM = Infant Milk Formula.

### Resistance to drying

**Figure 4:**
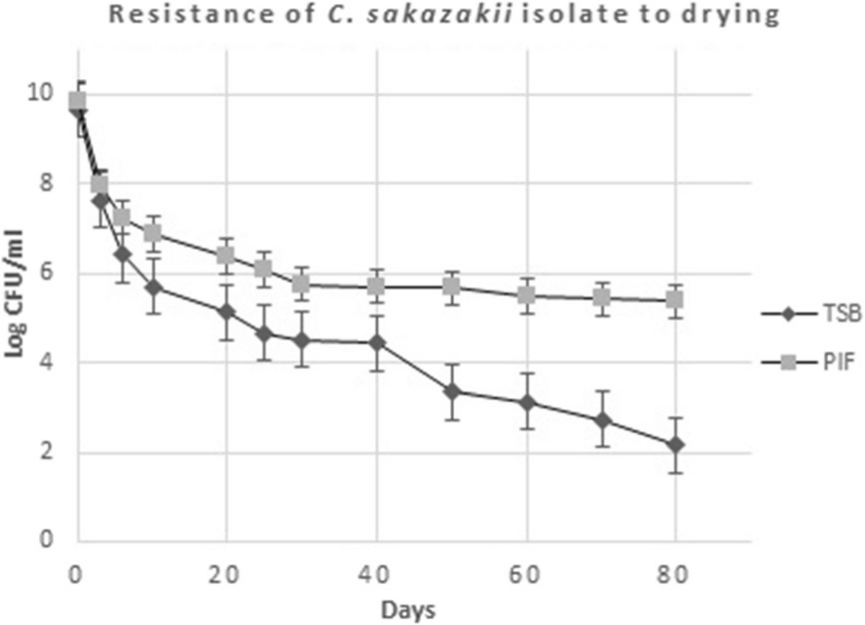
**Resistance of isolated** ***C. sakazakii*** **to drying.**

Overall, *C. sakazakii* isolates grown and dried in IF showed significantly (P <0.05) better survival during drying than grown and dried in TSB. Reductions were significantly higher in TSB than IF after 10 (P <0.05) and 80 (P <0.01) days storage at 30°C, but not at 20 days (P = 0.059). After 3 days, reductions in TSB were 2.04 log, but after 20 days, reductions increased to 4.54 log. In comparison, after 3 days in IF, reductions were 1.89 log, whereas after 20 days, reductions only increased to 3.45 log (Figure 4). There appeared to be a positive relationship between heat resistance and dehydration stress resistance of each strain. Meanwhile, survival of strong biofilm formers was not significantly different (P >0.05) than the survival of weak biofilm formers during drying in either TSB or IF.

### Resistance to low pH

**Figure 5:**
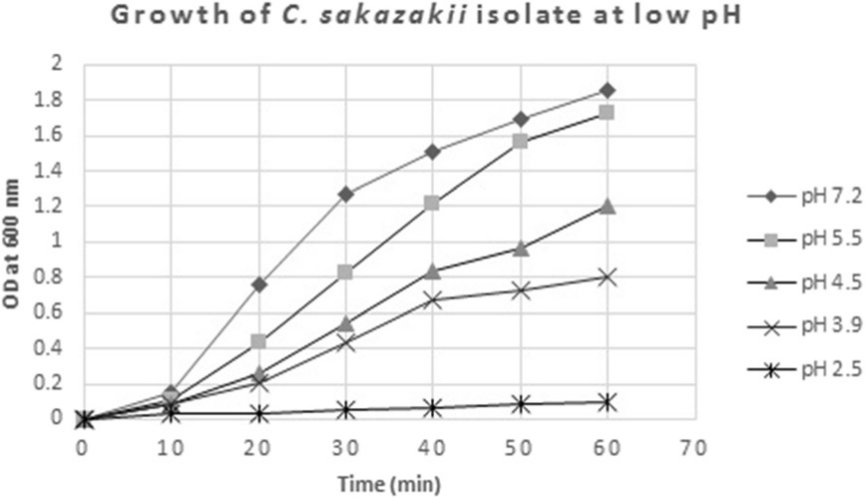
**Resistance of isolated** ***C. sakazakii*** **to low pH.**

Overall, the mean OD_600_ of *C. sakazakii* strains was highest for pH 7.2, which was not significantly (P >0.05) higher than the mean OD_600_ for pH 5.5. The mean OD_600_ at pH 4.5 was significantly (P <0.05) lower than the mean OD_600_ at pH 5.5, but difference between OD_600_ of pH 4.5 and 3.9 was not significant (P >0.05). The isolates were unable to survive at very low pH (2.5) (Figure 5).

### Resistance to osmotic stress

**Figure 6:**
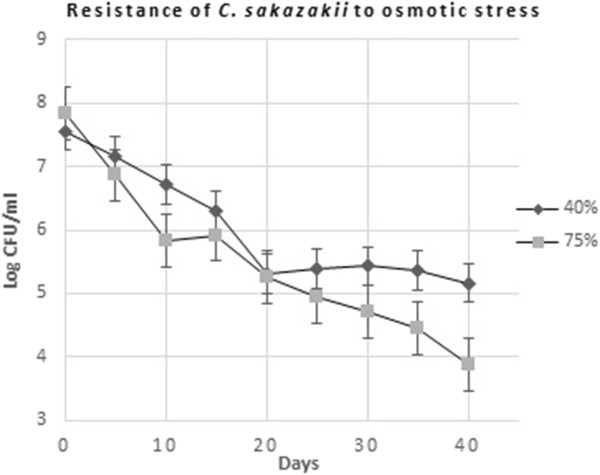
**Osmotic stress resistance of** ***C. sakazakii*** **isolate.**

In BHI with 40% sorbitol (aw 0.934), after 40 days, the number of cells had decreased with ca 2 log and in case of BHI with 70% sorbitol (aw 0.811), ca 4 log reduction was observed after 40 days (Figure 6).

## Discussion

Among the 54 food samples, 6 samples were contaminated with *Cronobacter sakazakii* (11.11%). A total of 6 *Cronobacter sakazakii* were isolated; 3 out of 15 milk powder samples, 1 out of 6 horlicks samples, 1 out of 10 biscuits samples and 1 out of 5 spices samples. The highest percentage of *Cronobacter sakazakii* isolates (50%) was found in milk powder. But a good number of samples were contaminated with other related organisms indicating lack of hygiene in production. These 6 isolates were identified as *Cronobacter sakazakii* on the basis of biochemical tests, sugar fermentation, SDS-PAGE of whole cell protein, plasmid profile and PCR of *Cronobacter* spp*.* specific genes (*esak*, *gluA*, saka, ERIC, BOX-AIR).

Table 2 summarizes gene profiling of isolated *C. sakazakii* strains along with *C. muytjensii* ATCC 51329 and *C. sakazakii* ATCC 29544. *esak* gene, which is most reliable for detection of *Cronobacter sakazakii* was found in 6 isolates those are phenotypically similar to *Cronobacter sakazakii*. Cawthorn *et al*. [13] also isolated *Cronobacter sakazakii* with esak gene and *gluA* gene. All the 6 isolate possess at least one or more of the four gene (*gluA* &*saka*) reported by many researchers to be present in *C. sakazakii*. Three strains (MP04.1, HR11.3 & BC52.2) possessed *saka* gene. *Cronobacter sakazakii* isolated by Hassan *et al*. [14] and Cawthorn *et al*. [13] also possessed *saka* gene.

Identity was confirmed by 16 s rDNA sequencing. Phylogenetically the *Cronobacter sakazakii* isolates produced 2 distinct clusters in the tree. Isolates MP04.1, M08.5 & SP62.1 in a cluster and isolates BC52.2, HR11.3 & MP10.2 in another cluster. Similar results have been found by Iversen *et al*. [15]. They showed sequence analysis based on 16S rDNA of 210 strains resulted in four clusters. The majority of strains were grouped in cluster 1 together with the *Enterobacter sakazakii* type strain, ATCC 29544.

All the isolates have 5 common bands in BOX-PCR fingerprint which supports this identity as of same species. These isolates also have significant differences in this BOX fingerprint which indicates that these isolates belong to different genotypes of *C. sakazakii* which was supported according to phylogenic results. These isolates were isolated from different food samples which may also contribution to this difference in BOX fingerprints [16]. ERIC-PCR is a well-used technique to genotype any bacteria. All of them were found to 2 or more than 2 to prominent bands. 16 biogroup has been reported and the existence of several genetic groups has been demonstrated based on 16S rRNA gene sequence analysis [17]. Variations in ERIC-PCR product also demonstrate that the 6 isolated *Cronobater sakazakii* strains belong to different genotypes.

All of the isolated *Cronobacter sakazakii* were susceptible to chloramphenicol, gentamycin and most of them were resistant to vancomycin, ampicillin, nitrofurantoin, penicillin, imipenem, In this study only one strain (SP62.1) was resistant to tetracycline and doxycycline. Nazarowec-White & Farber [18] & Mofokeng *et al*. [19] who isolated *Cronobacter sakazakii* resistant to ampicillin, imipenem, and neomycin. Two isolate were ESBL positive indicating emergence of ESBL in food-borne *Cronobacter sakazakii* isolates. In a previous study by Townsend et al. [20], ESBL positive *Cronobacter sakazakii* has also been reported.

Virulence factors are important to understand bacterial pathogenesis and interactions of them with the host. Virulence properties may also aid to select novel targets in drug and vaccine development [21]. These virulence factors make some members of the normal flora component to cause an infection by overcoming the mechanisms [22]. *Cronobacter* display differences in pathogenicity and may have different virulence factors [23]. Three isolates (MP08.5, BC52.2 & SP62.1) were found to be able to produce extracellular protease enzyme. According to Lockwood *et al*. [24], an important virulence factor of the *Cronobacter* species is the production of protease enzymes. In this study *Cronobacter sakazakii* isolate BC52.2 & SP62.1 was found to possess metaloprotease specific zpx gene. 50% isolated (MP04.1, MP10.2 & SP62.1) strains were able to bind congo red indicating expression of curli fimbriae. These stimulate the host inflammatory response and contribute to persistence within the host, and are also required for formation of biofilms [25]. In this study isolated strains which were capable of congo red binding, were also biofilm producers. Two strains of isolated *Cronobacter sakazakii* (MP08.5 & MP10.2) were

β hemolytic. All the 6 isolated strains gave haemagglutination with 2% and 4% human type O Rh positive blood suspension. Mannose-resistant haemagglutination (MRHA) were shown 100% (6/6) isolates. Mannose -inhibit the bacterial colonization of mammalian cell membrane. MRHA resistant *Cronobacter sakazakii* can easily cross the cell membrane defenses of the body. Surface hydrophobicity of bacteria is an important virulence factor. 4 out of 6 isolated *Cronobacter sakazakii* strains had minimum cell surface hydropohobicity at 3% ammonium sulphate solution. All the isolates were able to produce siderophore, a virulence factor that may be associated with ability of bacteria to causes necrotizing enterolcolitis [26].

Only one isolate possessed *ompA* gene. The outer membrane protein A (OmpA) of *Cronobacter* spp. is involved in the colonization of the gastrointestinal tract and invasion of human intestinal epithelial and brain endothelial cells, as well as subsequent survival in blood to cause meningitis [11, 12]. *Cronobacter sakazakii* possessed zpx gene *zpx* gene codes for proteolytic enzyme [27]. All isolated strains showed serum tolerance. Pathogenic microorganisms cause invasive infections have evolved strategies to protect themselves against the bactericidal action of the serum/complement. The outer membrane protein Omp A contributes significantly to the survival of the *Cronobacter sakazakii* in the blood. The outer membrane protease of *Cronobacter sakazakii* activates plasminogen and mediates resistance to serum bactericidal activity.

Isolated *Cronobacter sakazakii* SP62.1 was strong biofilm producer. Strains with higher motility had strong biofilm production ability. Similar study has been observed by Iversen *et al*. [28] who found some strains of *Cronobacter* spp. are able to form biofilms on glass, stainless steel, polyvinyl chloride (PVC), polycarbonate, silicone, and enteral feeding tubes. Enterotoxins were found in all 6 isolated *Cronobacter sakazakii* strains. They were about 66 KDa along with three other higher molecular weight proteins. These may be other enterotoxin proteins Additionally, *Cronobacter* strains have been found to produce an enterotoxin [29]. The enterotoxin of 66 KDa had been purified by Rhagav & Aggrawal [30]. The isolates showed significant serological cross reactivity with different serotypes of *Salmonella*, *Shigella boydii* and *Vibrio cholerae*. Isolated MP10.2 of six strains gave agglutination with 13 commercially available antisera except *Shigella boydii* polyvalent 3(2–15). Both M4 and M8 strains gave agglutination with Salmonella polyvalent o group A-S , *Shigella boydii* polyvalent 3(12–13), strains M4 gave agglutination with *Shigella sonnei* phase 1&2 and *Vibrio cholerae* O1 polyvalent, Strains M8 gave agglutination with *Shigella boydii* polyvalent 3(2–15).

4 (66.67%) isolated *Cronobacter sakazakii* were able to grow at 10% NaCl concentration.MP04.1 & HR11.3 both were able to grow at 7%; growth reduced at 8% NaCl concentration. All of the isolated (6/6) *Cronobacter sakazakii* were able to grow 5% bile salt concentration. Bile is an important antimicrobial component of the human digestive system. If the membrane is compromised by bile salts, then the toxic effects could be conveyed to the DNA, leading to extensive damage in the form of reactive oxygen species. This would lead to the cessation of replication and eventually cell death [31]. The isolates showed tolerance to different stresses such as temperature, dryness, low pH and osmotic stress.

## Conclusion

*Cronobacter sakazakii* is an emerging pathogen, often transmitted through powdered milk and responsible for a series of infections, some of which with potential fatal outcomes, in a particular segment of the population. This study reveals that food samples of Bangladesh is contaminated with *Cronobacter sakazakii* and combined effort showed be formulated to reduce the risks posed by this bacterium.

## Methods

### Sample collection

Fifty four (54) different foods from different manufacturers were purchased from retail stores across Dhaka, Bangladesh. The samples were 15 milk powder, 6 horlicks, 6 honey, 6 chutney, 6 chocolates, 10 biscuits and 5 spices.

### Isolation of *Cronobacter*spp

The procedure of FDA [32] for detection, isolation and identification of *Cronobacter sakazakii* in food samples was followed. All of the food samples were added to buffered peptone water (BPW) in 1:10 (10 g of samples/ 90 ml BPW) for pre-enrichment and incubated 18–24 hrs at 37°C. Then pre-enriched samples were added to Cronobacter screening broth (CSB Broth) or Enterobacteriaceae enrichment (EE) broth (Oxoid Ltd., UK) in 1:10 which contains bile salt and brilliant green to suppress the growth of non Enterobacteriaceae and incubated for 18–24 hrs at 37°C. Two plates of violet red bile glucose (VRBG) agar were inoculated (0.1 ml) by streaking method from the CSB broth or EE broth culture. Another loopful of the suspension was streaked on VRBG agar. The plates were incubated for 18–24 hrs at 37°C. Five colonies of the red or purple colonies surrounded by purple halo were examined morphologically and streaked on TSA; plates were incubated for 24–72 hrs at 25°C.

### Identification

*Cronobacter sakazakii* were presumptive confirmed by growing them on HicromeEnterobacter sakazakii agar [33]. Oxidase and catalase test, citrate utilization test, methyl red, Voges-Proskauer, Kligler’s iron agar (KIA), nitrate reduction test, arginine decarboxylase, esculin hydrolysis, gelatin hydrolysis and indole production. Rhamnose, xylose, trehelose, ducitol, arabinose, salicin, mannitol, sucrose, lactose, malibiose, sorbitol, maltose, cellubiose and glucose were used in carbohydrate fermentation assay. Presumptive *Cronobacter sakazakii* positive isolates were streaked on MUG MacConkey agar containing 4 Methylumbelliferyl β-D-glucuronide (HiMedia, India) a substrate which upon being metabolized forms yellow colonies that fluoresce under UV light [34].

### Whole cell protein profile

To determine the protein profiles extracted with extraction buffer, SDS-PAGE analysis (Bio- Rad, USA) was done followed by coommassie blue staining. Centrifuge (12000 rpm for 10 min) 1.5 ml of the culture grown on TSB. Discarded all medium and pellet was washed with Phosphate buffer saline (PBS). Centrifuge at 12000 rpm for 5 min. Supernatant was discarded and pellet was suspended in 1 ml extraction buffer (10% Glycerol; 2% SDS; 0.05MTris; PH 6.8) and boiled for 5 min in water bath. Centrifuge at 12000 rpm for 15 min after boiling. Supernatant is separated by 0.45 μm filter with the help of syringe (5 ml).

### Plasmid profile

Plasmid DNA was extracted from presumptive isolates of *Cronobacter sakazakii* byPureLink® Quick Plasmid Miniprep Kit (Invitrogen™) through supplied procedure. Plasmid DNA was separated by electrophoresis 0.7% agarose gels in a Tris-boric EDTA (TBE) buffer at room temperature at 90 volt for 1 hr. The gel was stained with 0.02% ethidium bromide for 10 min at room temperature and distained with distilled water for 10 min. DNA bands were visualized photograph was taken with UV transiluminator.

### Chromosomal DNA extraction

Then total DNA was extracted by Accuprep® genomic DNA extraction kit Cat. No.: K-3032 by the supplied procedure.

### Molecular characterization

For molecular identification of the *C. sakazakii* PCR amplication was performed with five primer pairs (Table 8). In PCR reaction *Cronobacter muetjensii* ATCC 51329 was used as the positive control. Reactions using primers Esakf/Esakr was optimized in a 50 μl reaction mixture consisting of 4 μl of the bacterial genomic DNA solution (50 ng), 5 μl PCR buffer, 3 μl MgCl_2_ (25 mM), 2 μl dNTPs (10 mM), 2 μl DMSO 99% 0.7 μl Taq DNA polymerase 143 5U/μl, 1 μl (100 nM each) primers and 31.3 μl nuclease free water. Reactions using primers EsgluAf/EsgluAr was optimized in a 50 μl reaction mixture consisting of 4 μl of the bacterial genomic DNA solution (50 ng), 5 μl PCR buffer, 3 μl MgCl_2_ (25 mM), 3 μl dNTPs (10 mM), 0.7 μl Taq DNA polymerase 5U/μl, 1 μl (100 nM each) primers and 32.3 μl nuclease free water. Reactions using primers Saka1a-F/Saka2b-R, ESSF/ESSR and ZpxF/ZpxR were optimized in a 50 μl reaction mixture consisting of 4 μl of the bacterial genomic DNA solution (50 ng), 5 μl PCR buffer, 3 μl MgCl_2_ (25 mM), 2μldNTPs (10 mM), 0.7 μl Taq DNA polymerase 5U/μl, 1 μl (100 nM each) primers and 33.3 μl nuclease free water. PCR products were analyzed using 1.5% (w/v) agarose gel electrophoreses in 0.5 × TBE buffer and a constant voltage of 90 V to confirm the presence of amplified DNA [35].

**Table 8:** **List of primer pair for PCR amplification**

Primer	Sequence 5' to 3'	Targeted site	Product size (bp)	Reference
Esakf/	GCTYTGCTGACGAGTGGCGG^a^	16S rDNA	929	[33]
Esakr	ATCTCTGCAGGATTCTCTGG			
EsgluAf/	TGAAAGCAATCGACAAGAAG^b^	*gluA*	1680	[34]
EsgluAr	ACTCATTACCCCTCCTGATG			
Saka 1a/	ACAGGGAGCAGCTTGCTGC^c^	V1g	952	[14]
Saka 2b	TCCCGCATCTCTGCAGGA	V3h		
ESSF/	GGATTTAACCGTGAACTTTTCC^d^	*ompA*	469	[35]
ESSR	CGCCAGCGATGTTAGAAGA			
Zpx F/	GAAAGCGTATAAGCGCGATTC^e^	*zpx*	94	[27]
Zpx R	GTTCCAGAAGGCGTTCTGGT			

^a&c^Running conditions; 94°C for 5 min; 35 cycles of 94°C for 1 min each; 60°C for 1 min; 72°C for 1.5 min; a final extension period of 5 min at 72°C.

^b^Running conditions; 94°C for 10 min; 35 cycles of 94°C for 30 sec each; 60°C for 1 min; 72°C for1.5 min; final extension period of 5 min at 72°C.

^d^Running conditions; 94°C for 5 min; 35 cycles of 94°C for 1 min each; 54°C for 45 sec; 72°C for1.5 min; final extension period of 5 min at 72°C.

^e^Running conditions; 94°C for 15 min; 35 cycles of 94°C for 1 min each; 58°C for 1 min; 72°C for1.5 min; final extension period of 10 min at 72°C.

### BOX-AIR PCR

Reactions using primer BOX-A1R (Table 9) was optimized in a 50 μl reaction mixture consisting of 4 μl of the bacterial genomic DNA solution (50 ng), 5 μl PCR buffer, 2 μl MgCl_2_ (25 mM), 2 μl dNTPs (10 mM), 0.7 μl Taq DNA polymerase 5U/μl, 2 μl (300 nM) primer and 34.3 μl nuclease free water [36, 37].

**Table 9:** **Primer sequence of BOX-AIR and ERIC1R/ERIC2**

Primer	Sequence 5’ to 3’	Reference
ERIC1R	ATGTAAGCTCCTGGGGATTCAC^f^	[36]
ERIC2	AAGTAAGTGACTGGGGTGAGCG	
BOX-A1R	CTACGGCAAGGCGACGCTGACG^g^	[37]

^f&g^Running conditions; 94°C for 8 min; 35 cycles of 94°C for 30 sec each; 55°C for 1 min; 72°C for 8 min; a final extension period of 10 min at 72°C.

### ERIC-PCR fingerprinting

Reactions using primers ERIC1R/ERIC2 (Table 9) was optimized in a 50 μl reaction mixture consisting of 4 μl of the bacterial genomic DNA solution (50 ng), 5 μl PCR buffer, 2 μl MgCl_2_ (25 mM), 2 μl dNTPs (10 mM), 0.5 μl Taq DNA polymerase 5U/μl, 1 μl (200 nM each) primers and 34.5 μl nuclease free water.

### 16S rDNA gene sequencing

Sequencing of partial 16S rDNA of the isolated *C. sakazakii* was performed with universal primers according to as described by Loffler *et al.* [38]. The purified cycle sequence product was analyzed by electrophoresis in the ABI-Prism 310 Genetic Analyzer (Applied Biosystems, USA). Raw sequence from automated DNA sequence was edited using Chromas2.33 software. After editing the sequence was saved as FASTA format for further analysis. The homology of the 16S rRNA gene sequences was checked with the 16S rRNA gene sequences of other organisms that had already been submitted to GenBank database using the BLAST (http://www.ncbi.nih.gov/Blast.cgi) algorithm.

### Phylogenetic analysis

Sequence alignment of 16S rDNA genes of isolated *Cronobacter sakazakii* and some other related species was performed with ClustalW using default matrix within MEGA version 5. Phylogenetic tree was inferred by the neighbor-joining method using the software MEGA version 5.0 package [39].

### Virulence properties of isolated *Cronobacter sakazakii*

Protease production, haemolytic activity, haemagglutination ability, cell surface hydrophobicity, congo red binding, resistance against blood serum was performed according to Fakruddin et al. [40]. Siderophore production was determined according to the method described by Payne [41].

### Antibiotic sensitivitiy

Cronobacter isolates were tested for their susceptibility to vancomycin (30 μg), ciprofloxacin (5 μg), ampicillin (10 μg), nitrofurantoin (300 μg), chloramphenicol (30 μg), penicillin G units (10 μg), tetracycline (30 μg), ampicillin (10 μg), imipenem (10 μg), doxycycline (30 μg),neomycin (10 μg), Amikacin (30 μg), gentamycin (10 μg) (Antibiotic disks were obtained from Emapol, Poland) using the Kirby-Bauer agar disc diffusion method [42] following CLSI guidelines [43].

### ESBL production

The combination disc method as described by Townsend et al. [20] was used to detect ESBL activity.

### Biofilm formation assay

Biofilm formation assays were performed following the method Danese *et al.* [44] with some modifications. The assays were performed twelve times. Biofilm measurements were made using the formula SBE = AB-CW/G in which SBE is the specific biofilm formation, AB is the OD490 nm of the attached and strained bacteria, CW is the OD490 nm of the control wells containing only bacteria-free medium (to eliminate unspecific or abiotic OD values), and G is the OD490 nm of cells growth in broth. The SBE values were classified into two categories: strong biofilm producers (SBF index > 1.00) and weak biofilm producers (SBF index > 1.00).

### SDS-PAGE analysis of enterotoxin of the isolates

The six isolated strains were cultured in brain heart infusion broth (Himedia, India) and subsequently screened for enterotoxin production in casminoacids yeast extract broth according to Raghav & Agarwal [30]. 50 ml of casamino acids yeast extract broth was inoculated (1%v/v) with an overnight culture of the most potently enterotoxic *C. sakazakii* and incubated at 30°C for 24 hrs. The culture was sub cultured into 500 ml of broth, which was then dispensed in 100 ml volumes into five 250 ml flasks. Each culture was incubated at 30°C on a rotary shaker operating at 160 rpm for 18 hrs then centrifuged at 14,000 rpm for 20 min. The supernatants were recovered and pooled, and the pellets were discarded. The supernatants were filtered through a 0.45 μm membrane filter. Protein was isolated from this supernatant by 50% ammonium sulphate; at step the sample was left for 10 min to ensure equilibrium and the centrifuged (5000 rpm for 30 min) to collect precipitated proteins. The final pellet was dissolved in 30 ml of 0.05 mol/L tris (pH 8.0). 25 μl aliquots will be loaded onto 12.5% bisacrylamide gel.

### Serological cross reactivity of the isolates

Commercially available agglunating serum (Remel, UK) such as Salmonella 2–0, Salmonella polyvalent o group A-S, Salmonella typhi O-Group D somatic antigen, Salmonella paratyphi A O Group A somatic antigen, Salmonella 9–0. *Shigella boydii* polyvalent 3(12–13), *Shigella boydii* polyvalent 1(1–6), *Shigella sonnei*phase 1&2, *Shigella flexneri* polyvalent (1–6, x&y), *Shigella boydii* polyvalent 2(7–11), *Shigella boydii* polyvalent 3(2–15), *Vibrio cholerae* O1 polyvalent, *Vibrio cholera*e inaba, *Vibrio cholera*e ogawa were used to agglutination of strains. 25 μl of normal saline was taken on a glass slide and bacterial colonies were emulsified in it to get a homogenous milky-white suspension (approximately 10^9^ cfu/ml). Then 10 μl of antisera was added to the bacterial suspension on the glass slide. The slide was rotated and macroscopic agglutination was observed within one minute [45].

### Stress tolerance

#### Salt tolerance test

Isolates were inoculated in TSB containing different NaCl concentration (3%, 5%.7%, 8%, and 10%) at 37°C for 24 hrs. Spectrophotometer readings were taken with an absorbance of 600 nm for each sample at 0 hr and 24 hrs.

#### Bile salt tolerance

Isolates were inoculated in TSB containing different bile salts concentration (1%, 2%.3%, 4% and 5%) at 37°C for 24 hrs. Spectrophotometer readings were taken with an absorbance of 600 nm for each sample at 0 hr and 24 hrs.

#### Thermotolerance

The thermotolerance of isolated *C. sakazakii* was determined by suspending 1 ml overnight culture in 20 ml of temperature equilibrated TSB and IFM in water baths between 54 and 62°C. At timed intervals 0.1 ml aliquots were transferred to 2 ml TSB at room temperature and the number of survivors determined [28]. The number of survivors at each temperature was plotted against time. The best fit-line was extrapolated and the *D* values were determined (-1/slope of the regression line). The *z* values were determined by plotting the calculated log *D* values against the corresponding temperatures (-1/slope of the regression line). Each single number is an average of three replicate experiments. The standard deviations of the *D* value and *z* values were calculated [46].

### Resistance to drying

The ability of the isolates to withstand drying was tested according to the procedure described by Breeuwer et al. [47]. Briefly, 50 μl aliquots of 24 h culture in TSB or IFM were placed in 12-well sterile polystyrene tissue culture plates (Corning Inc., Corning, NY, USA) and allowed to air dry in a 30°C incubator. The original culture was enumerated and reported as CFU ml^-1^ on day 0. On periodic interval up to 80 days, the inoculum dried in the incubator was reconstituted in 1 ml of sterile peptone water, and appropriate dilutions were plated on TSA to determine the survivors.

### Resistance to low pH

Test strains were cultured for 12 h in TSB to late exponential phase. TSB test media were adjusted to the target pH, 4.5, 4.3, 4.1 and 3.9. The growth of the test strains in the test media adjusted to different pH levels was measured by the method as described by Dancer et al. [48].

### Resistance to osmotic stress

Stationary or exponential phase cell suspensions were added to BHI +75% (w/v) sorbitol (a_w_ 0.811) and BHI +40% sorbitol (a_w_ 0.934) to obtain an initial level of ca 10^7^ cells per ml. The suspensions were incubated at 25°C and at regular intervals samples were taken for enumeration of survivors [47].

## Electronic supplementary material

Additional file 1: ***Cronobacter sakazakii*** **isolates in culture media.** (DOCX 323 KB)
