# Supplementary material for: Stress tolerant virulent strains of Cronobacter sakazakii from food
Source: Biol Res. 2014 Nov 25;47(1):63. doi: 10.1186/0717-6287-47-63 (PMC4335510; doi:10.1186/0717-6287-47-63)
Supplement: Supplementary file 1 — Additional file 1: Cronobacter sakazakii isolates in culture media. (DOCX 323 KB) [file 40659_2014_74_MOESM1_ESM.docx]

***Cronobacter sakazakii* isolates in culture media**

**
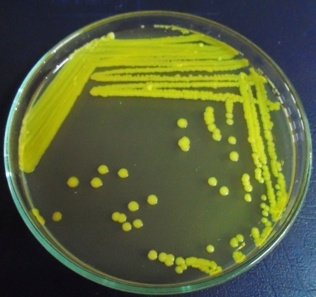
**

Figure 1: Characteristic yellow & water like yellow colonies on TSA

**
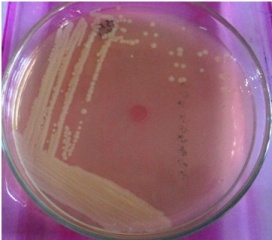
**

Figure 2: Characteristic Pink colonies on VRBG


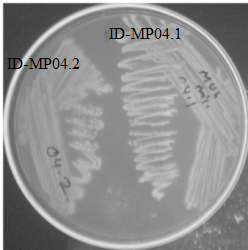


Figure 3: Fluorescence (250 nm) of isolate on MUG MacConkey agar


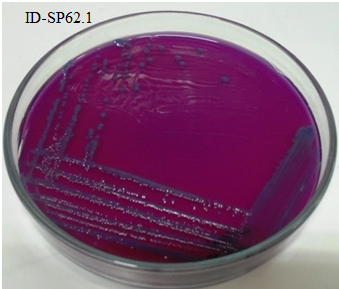


Figure 4: Blue green colonies of isolate on Hicrome Enterobacter sakazakii agar
